# Chronic Nonbacterial Osteomyelitis in a Young Child: A Case Report of a Diagnostic Challenge Mimicking Malignancy

**DOI:** 10.7759/cureus.85684

**Published:** 2025-06-10

**Authors:** Alexander N Fahmy, Margaux Everingham, Chris Kim, Ameet Kumar

**Affiliations:** 1 Pediatrics, Drexel University College of Medicine, Philadelphia, USA; 2 Cardiology, University of Pittsburgh Medical Center, Harrisburg, USA; 3 Pediatrics, University of Pittsburgh Medical Center, Harrisburg, USA

**Keywords:** chronic non-bacterial osteomyelitis, osteosarcoma, pediatric musculoskeletal disorders, pediatric oncology, pediatric rheumatology

## Abstract

Chronic nonbacterial osteomyelitis (CNO) is a rare, autoinflammatory bone disorder that primarily affects children and adolescents. It typically presents with localized bone pain and lacks systemic symptoms, making diagnosis difficult. Radiologic features often mimic malignant or infectious etiologies, contributing to delays in diagnosis and the use of unnecessary invasive procedures.

We report the case of a 5-year-old female patient with a history of sickle cell trait who presented with intermittent left arm pain. The initial physical examination was unremarkable, and symptoms were attributed to benign musculoskeletal causes. However, persistent symptoms over a five-month period prompted further evaluation. Radiographs revealed a permeative lesion in the humerus with areas of sclerosis and lucency, along with a multilayered periosteal reaction. Magnetic resonance imaging (MRI) showed an enhancing intraosseous lesion with cortical breakthrough and periosteal edema, raising concerns for malignancy, including osteosarcoma and Ewing sarcoma.

Laboratory studies showed elevated erythrocyte sedimentation rate (ESR) and mildly increased C-reactive protein (CRP), but other results were unremarkable. Given the imaging findings and concerning differential diagnoses, a computed tomography (CT)-guided bone biopsy was performed. Histopathology confirmed chronic inflammatory changes consistent with CNO, and no evidence of neoplasia or infection was found. Whole-body magnetic resonance imaging revealed no other lesions. The patient was referred to pediatric rheumatology and began treatment with methotrexate, selected due to the lesion size and risk of pathologic fracture. The patient showed marked clinical improvement and returned to baseline function within six months.

CNO, also referred to as chronic recurrent multifocal osteomyelitis in its multifocal form, remains underrecognized due to its vague symptoms and radiologic similarity to more ominous pathologies. Biopsy often plays a critical role in excluding malignancy. CNO can be unifocal or multifocal and is associated with other autoimmune conditions such as inflammatory bowel disease, psoriasis, and juvenile idiopathic arthritis. Given the patient’s family history of ulcerative colitis, long-term monitoring is warranted. The condition predominantly affects female individuals and typically presents between the ages of 7 and 12. Treatment focuses on anti-inflammatory and immunomodulatory therapies. While nonsteroidal anti-inflammatory drugs are first-line agents, second-line therapies such as methotrexate, tumor necrosis factor inhibitors, corticosteroids, and bisphosphonates may be needed in more severe or refractory cases.

CNO is a rare but clinically significant pediatric condition that can mimic malignancy and cause considerable diagnostic anxiety. Awareness among clinicians, particularly regarding atypical presentations such as unifocal disease in very young children, is essential to avoid unnecessary procedures and initiate early effective treatment. A multidisciplinary approach is often required for accurate diagnosis and optimal management.

## Introduction

Chronic nonbacterial osteomyelitis (CNO) is a rare, autoinflammatory bone disorder that primarily affects children and adolescents. It typically presents with localized bone pain and lacks systemic symptoms, making diagnosis difficult. While there are no universally accepted diagnostic criteria, the Jansson criteria [1] are frequently used and include clinical symptoms, radiologic findings, exclusion of infection or malignancy, and in some cases, histopathologic confirmation. Radiologic features often mimic malignant or infectious etiologies, contributing to delays in diagnosis and the use of unnecessary invasive procedures. The disorder can present as unifocal or multifocal bone pain, frequently involving the metaphyses of long bones but also affecting the spine, pelvis, and clavicle. The clinical course can be intermittent or chronic, with episodes of exacerbation and remission, sometimes extending into adulthood.

CNO presents a significant diagnostic challenge due to its nonspecific clinical and radiologic features, which can closely mimic malignant bone lesions like osteosarcoma or Ewing sarcoma. These lesions may exhibit mixed sclerotic and lytic changes, periosteal reaction, and bone marrow edema, often prompting extensive diagnostic workups to exclude malignancy. However, certain radiologic patterns, such as symmetric multifocal involvement and the absence of a soft tissue mass, can help differentiate CNO from malignant conditions. Histopathological confirmation via bone biopsy is often required, revealing chronic inflammation without evidence of infection or neoplastic cells.

Epidemiologically, CNO is rare, with a recent study estimating an incidence of approximately 0.65 per 100,000 children under 16 years of age in the UK and the Republic of Ireland [2], and it is more commonly observed in girls, with a peak onset between 8 and 10 years of age. It is also associated with other autoimmune and autoinflammatory disorders, including inflammatory bowel disease (IBD), psoriasis, and juvenile idiopathic arthritis (JIA), further complicating the clinical picture. This overlap emphasizes the need for comprehensive clinical assessment and ongoing monitoring. This case report aims to highlight the diagnostic challenges of CNO, emphasizing the importance of early recognition and multidisciplinary management to prevent long-term skeletal damage and unnecessary interventions.

## Case presentation

A 5-year-old female patient presented with a three-month history of intermittent left arm pain, described as a deep, throbbing ache that worsened at night and was partially relieved by non-steroidal anti-inflammatory drugs (NSAIDs). Initially attributed to benign causes such as growing pains, the pain gradually intensified, resulting in functional limitations and prompting further evaluation. Physical examination revealed no deformity, redness, warmth, or swelling, and her neurovascular status was intact. There were no systemic symptoms like fever, weight loss, or fatigue, which might have suggested a more aggressive or systemic process.

**Figure 1:**
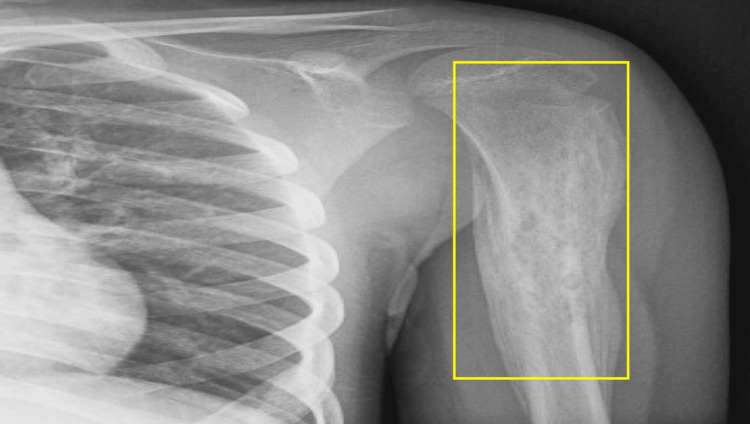
X-ray left humerus showing permeative mixed sclerosis and lucency and an extensive multilayered periosteal reaction

Initial radiographs (Figure 1) demonstrated a permeative lesion with mixed sclerosis and lucency in the proximal diaphysis of the humerus, accompanied by extensive multilayered periosteal reaction, raising concern for malignancy.

**Figure 2:**
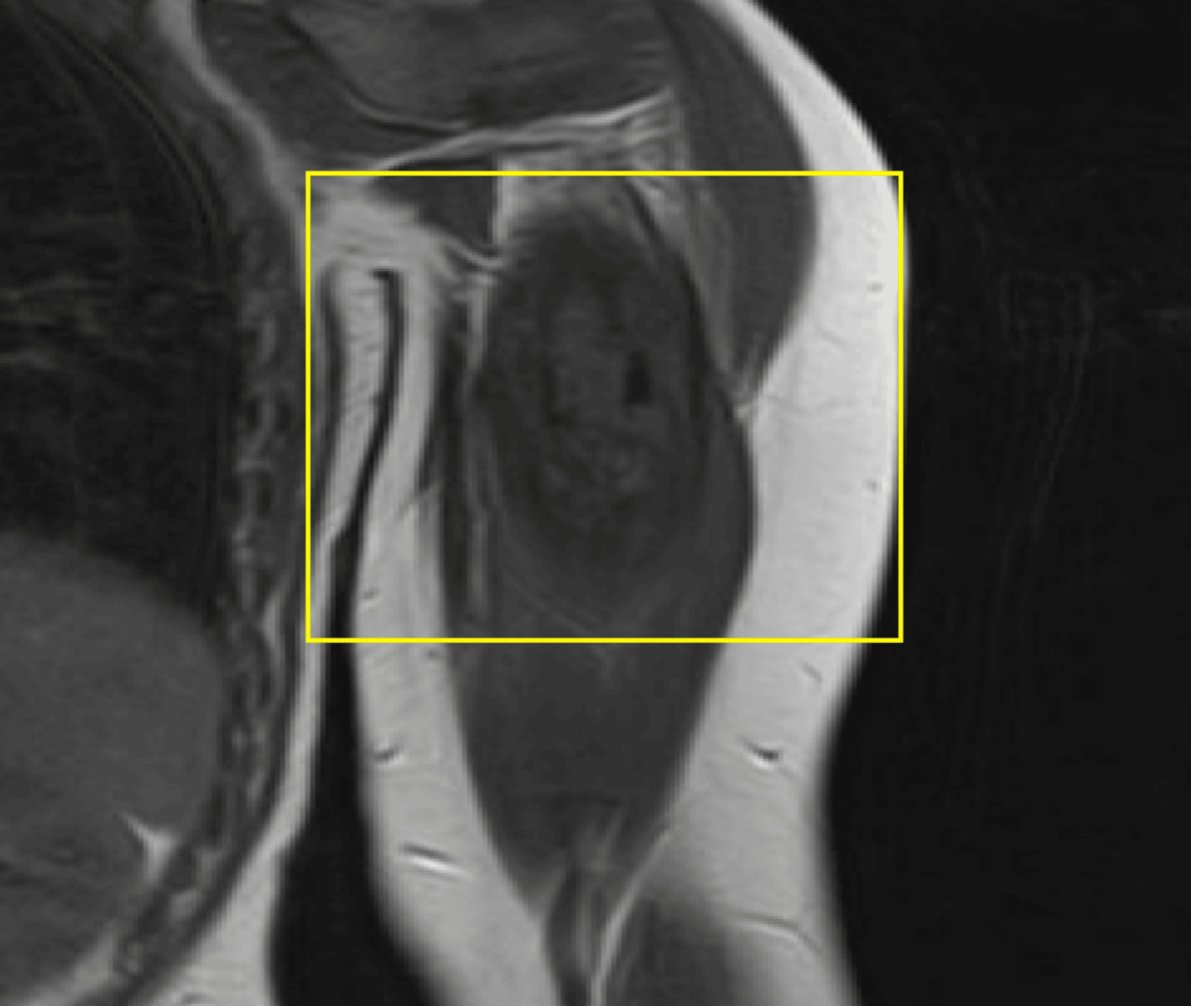
Intraosseous MRI showing enhancing destructive mass arising in the proximal diaphysis of the humerus with significant surrounding periosteal edema, abnormal cortical breakthrough, and periosteal reaction

MRI (Figure 2) confirmed a destructive intraosseous mass with significant periosteal edema and abnormal cortical breakthrough, findings often seen in aggressive bone lesions.

Laboratory tests showed elevated erythrocyte sedimentation rate (45 mm/hr) and mildly increased C-reactive protein (0.77 mg/dL (Table 1), indicating inflammation but lacking specificity for malignancy. At this stage, the differential diagnosis included Ewing sarcoma, osteosarcoma, leukemia, and Langerhans cell histiocytosis. The family became concerned due to the possibility of a malignancy based on MRI findings.

**Table 1:** Laboratory results and inflammatory markers ESR: erythrocyte sedimentation rate; CRP: C-reactive protein; mg/dL: milligrams per deciliter; mm/hr: millimeters per hour

Test	Patient result	Reference range	Units
C-reactive protein (CRP)	0.77	< 0.5	mg/dL
Erythrocyte sedimentation rate (ESR)	45	0–20	mm/hr

Given these concerning imaging findings, a CT-guided bone biopsy was performed to exclude malignancy, revealing chronic inflammatory changes without evidence of infection or neoplastic cells, consistent with CNO. This diagnosis shifted the treatment approach, and methotrexate was initiated as first-line therapy due to the lesion’s size, risk of pathologic fracture, and unresponsiveness to NSAIDs at initial presentation. The patient demonstrated significant clinical improvement, returning to baseline function within six months, underscoring the importance of recognizing CNO in the differential diagnosis of pediatric bone pain. No follow-up imaging was performed as the pain was appropriately controlled, and there were no concerns of multifocal disease spread.

## Discussion

CNO, also referred to as chronic recurrent multifocal osteomyelitis (CRMO) in its multifocal form, is an autoinflammatory bone disorder characterized by sterile bone inflammation. Although first described in the 1970s, CNO remains underrecognized, often due to its insidious onset, nonspecific clinical presentation, and radiologic features that mimic more serious conditions such as malignancies or infectious osteomyelitis [3,4].

The hallmark symptom of CNO is localized bone pain, which may be intermittent or persistent and typically lacks systemic symptoms. When present, systemic features may include low-grade fever, fatigue, or elevated inflammatory markers such as erythrocyte sedimentation rate (ESR) and C-reactive protein (CRP) [5]. These findings often trigger workups for malignancy or infection. Radiologically, lesions are usually metaphyseal and may demonstrate lytic or sclerotic changes, periosteal reaction, and bone marrow edema, which are features that can resemble those seen in Ewing sarcoma or bacterial osteomyelitis [6]. However, certain imaging characteristics, such as the absence of a soft tissue mass and the presence of symmetric or multifocal lesions in CNO, can help distinguish it from these malignancies.

Histologic evaluation via bone biopsy is often necessary to exclude malignancy and confirm the diagnosis of CNO, particularly in cases with unifocal lesions or atypical presentations [7]. In this case, the biopsy revealed chronic inflammatory changes without evidence of neoplastic cells or infection, supporting the diagnosis of CNO. Given the absence of malignant cells and the chronic nature of inflammation, the decision to initiate methotrexate was influenced by the lesion’s size, the risk of pathologic fracture, and the need for a more aggressive anti-inflammatory approach.

CNO is associated with other autoimmune and autoinflammatory disorders, including inflammatory bowel disease (IBD), psoriasis, and juvenile idiopathic arthritis (JIA) [8]. This overlap emphasizes the need for comprehensive clinical assessment and ongoing monitoring. Given the patient's family history of ulcerative colitis, clinicians should remain alert to possible gastrointestinal or dermatologic symptoms in the future.

Epidemiologically, CNO primarily affects children and adolescents, with a peak incidence between 7 and 12 years of age. It shows a significant female predominance, occurring more than twice as often in girls as in boys [9]. The pathogenesis is not fully understood but is believed to involve dysregulated innate immunity, with genetic studies implicating pathways related to cytokine signaling and inflammasome activity [10].

Management focuses on reducing inflammation and preventing long-term skeletal damage. Nonsteroidal anti-inflammatory drugs (NSAIDs) are the first-line treatment and are effective in many patients. In those with poor response or more severe disease, second-line options include corticosteroids, tumor necrosis factor (TNF) inhibitors (e.g., etanercept, adalimumab), bisphosphonates, and disease-modifying antirheumatic drugs (DMARDs) such as methotrexate or sulfasalazine [11,12]. Early recognition and treatment are associated with improved clinical outcomes and reduced risk of complications, including growth disturbance and chronic pain.

In summary, CNO is a challenging diagnosis requiring a high index of suspicion. A multidisciplinary approach that often involves rheumatology, radiology, pathology, and at times, gastroenterology or dermatology, is essential for accurate diagnosis and management. Increasing clinician awareness of this condition is crucial to prevent unnecessary interventions and ensure the timely initiation of appropriate therapy.

## Conclusions

This case highlights the diagnostic challenges of differentiating CNO from malignant bone tumors in pediatric patients. The overlapping clinical and radiologic features can lead to misdiagnosis and unnecessary invasive interventions. A systematic approach that includes thorough clinical assessment, imaging interpretation, and when needed, histopathologic evaluation is essential. Recognizing CNO as a potential malignancy mimic allows for earlier diagnosis, appropriate management, and the prevention of overtreatment and long-term complications.
